# Treating infants with *frigg*: linking disease aetiologies, medicinal plant use and care-seeking behaviour in southern Morocco

**DOI:** 10.1186/s13002-016-0129-4

**Published:** 2017-01-13

**Authors:** Irene Teixidor-Toneu, Gary J. Martin, Rajindra K. Puri, Ahmed Ouhammou, Julie A. Hawkins

**Affiliations:** 1 Section of Ecology and Evolutionary Biology (EEB), Harborne Building, School of Biological Sciences, University of Reading, Whiteknights, Reading, RG6 6AS UK; 2 Global Diversity Foundation, Marrakech, Morocco; 3 Centre for Biocultural Diversity, School of Anthropology and Conservation, University of Kent, Canterbury, Kent CT2 7NR UK; 4 Department of Biology, Laboratory of Ecology and Environment, Regional Herbarium MARK, Faculty of Sciences Semlalia, Cadi Ayyad University, PO Box 2390, Marrakech, 40001 Morocco

**Keywords:** Southern Morocco, High Atlas, Marrakech, *ferragga*, Medicinal plants, Disease aetiologies, Childhood ailments

## Abstract

**Background:**

Although most Moroccans rely to some extent on traditional medicine, the practice of *frigg* to treat paediatric ailments by elderly women traditional healers known as *ferraggat*, has not yet been documented. We describe the role of these specialist healers, document the medicinal plants they use, and evaluate how and why their practice is changing.

**Methods:**

Ethnomedicinal and ethnobotanical data were collected using semi-structured interviews and observations of medical encounters. Information was collected from traditional healers, namely *ferraggat*, patients, herbalists and public health professionals. Patients’ and healers’ narratives about traditional medicine were analysed and medicinal plant lists were compiled from healers and herbalists. Plants used were collected, vouchered and deposited in herbaria.

**Results:**

*Ferragat* remain a key health resource to treat infant ailments in the rural High Atlas, because mothers believe only they can treat what are perceived to be illnesses with a supernatural cause. *Ferragat* possess *baraka*, or the gift of healing, and treat mainly three folk ailments, *taqait, taumist* and *iqdi*, which present symptoms similar to those of ear infections, tonsillitis and gastroenteritis. Seventy plant species were used to treat these ailments, but the emphasis on plants may be a recent substitute for treatments that used primarily wool and blood. This change in *materia medica* is a shift in the objects of cultural meaningfulness in response to the increasing influence of orthodox Islam and state-sponsored modernisation, including public healthcare and schooling.

**Conclusions:**

Religious and other sociocultural changes are impacting the ways in which *ferraggat* practice. Treatments based on no-longer accepted symbolic elements have been readily abandoned and substituted by licit remedies, namely medicinal plants, which play a legitimisation role for the practice of *frigg*. However, beliefs in supernatural ailment aetiologies, as well as lack or difficult access to biomedical alternatives, still underlie the need for specialist traditional healers.

**Electronic supplementary material:**

The online version of this article (doi:10.1186/s13002-016-0129-4) contains supplementary material, which is available to authorized users.

## Background

Herbal remedies used in traditional medicine are the primary health care resource in many rural communities around the world [1, 2]. Traditional medicine is a dynamic system that encompasses the “knowledge, skills and practices based on the theories, beliefs and experiences indigenous to different cultures that are used to maintain health, as well as to prevent, diagnose, improve or treat physical and mental illness” [3]. The availability of biomedicine (defined here as the medical system based on western scientific principles), often perceived as a symbol of modernity, development and globalization in the non-Western world [4, 5], alters care-seeking behaviour and often displaces traditional medicines [6–8]. However, traditional medicine continues to be used because of its effectiveness, lack of modern medical alternatives, high cost of biomedical services, long distance to public health facilities, cultural preferences or a combination of these factors [9, 10].

Moroccans may use traditional and biomedicine simultaneously, and consider them compatible [11]. Moroccan ethnomedicine is itself a pluralistic system, blending Prophetic and Galenic humoral medicine [12]. Since medieval times, indigenous Berber medicine has incorporated aspects of oriental Arabic medicine, as well as Al-Andalusian and Sub-Saharan knowledge [13]. Classification and treatment of illnesses is based on the “hot/cold” dichotomy derived from humoral medicine, but also on personalistic aetiologies such as the evil eye (*lʕin*), sorcery (*suhur*) or intervention of spirits (*jnûn*) [12]. Whereas naturalistic causes, those that are physical, chemical or pathological, are more often treated with medicinal plants by herbalists (*attar, achaba*) and midwifes (*qblat*), personalistic or supernatural causes are treated ritualistically by holy men and women (*cherif, chorfa*) or Quran experts (*fqih*) [12, 14, 15]. However, minor ailments are treated at home by herbal remedies; popular knowledge of medicinal plants is widespread in both rural and urban Morocco (see for example [16–18]). Here we report on traditional healers that practice in southern Morocco called *ferraggat* that whilst treating ailments caused by supernatural causes, use medicinal plants as their main treatment.

*Ferraggat* (*ferragga* in singular) are not midwifes (*qblat*) but specialize in treating children and women’ illnesses. *Ferraggat* are consulted by lay people of all social backgrounds [19]; they are invariably women, normally elderly, who often use a mix of medicinal plants called *frigg* in their treatments. The word *frigg* is also used to refer to the treatment itself. Despite their ubiquity as traditional healers in urban and rural southern Morocco, they have been overlooked by the most important references on Moroccan medical anthropology and ethnobotany [11, 13, 20]. The context in which *ferraggat* work is one of partial or limited access to public health care. In 2015, there were approximately 19.8 under-five deaths per 1000 live births in Morocco (mortality rate of 27.6) with pneumonia, injuries and diarrhoea as the leading causes of death [21]. Although much effort has been put in Morocco towards establishing a national health system to improve health standards and child mortality has decreased considerably in the last decades, the system still has insufficient human and material resources, an uneven geographic distribution of health coverage in detriment of rural areas, and minimal insurance benefits [22].

Traditional medicines have been considered highly symbolic; thus most elements of medicine are meaningful, and meaning can have physiological effects, triggering biological responses in sick people [23]. Moerman and Jonas [23] argue that meaning plays a key role in understanding effectiveness of traditional medicines, which are used in culture-specific contexts. Cultural constructs of efficacy and concepts about health and illness underlie any physiological response to meaning [23, 24]. However, phylogenetically related medicinal plants are selected across cultures, suggesting pharmacological efficacy of herbal remedies [25, 26]. Studies identifying medicinal plants are common in the ethnobotanical literature, but few consider the illness explanatory models in which they are used [27, 28]. Nevertheless, there are examples in the literature where medicinal plant use is presented in an ethnographic context and described alongside ceremonial, ritualistic treatments [29–31]. These studies stress that both the experience of illness and its treatments need to be understood in their cultural and social contexts. In the case of Morocco, studies either emphasise botanical identifications of medicinally-used plant species [16, 17] or the links between ailment aetiologies and healthcare seeking behaviour [11, 12, 14, 32].

Given the scarcity of literature describing the *ferraggat* and their practices, the initial aim of this study was to fill this knowledge gap, understanding how knowledge about *frigg* is learned and transmitted. We describe the ailments treated by *ferraggat* from an emic perspective and build a basic explanatory model [28], focusing on the symptoms, known causes, therapies and prognosis. We also aimed to understand the selection of medicinal plants used in *frigg*, linking the treatment with folk healing specialists, popular explanatory models and care-seeking behaviour, in a sociocultural context of recent modernisation and increasing availability of biomedical resources.

### Study sites

**Fig. 1:**
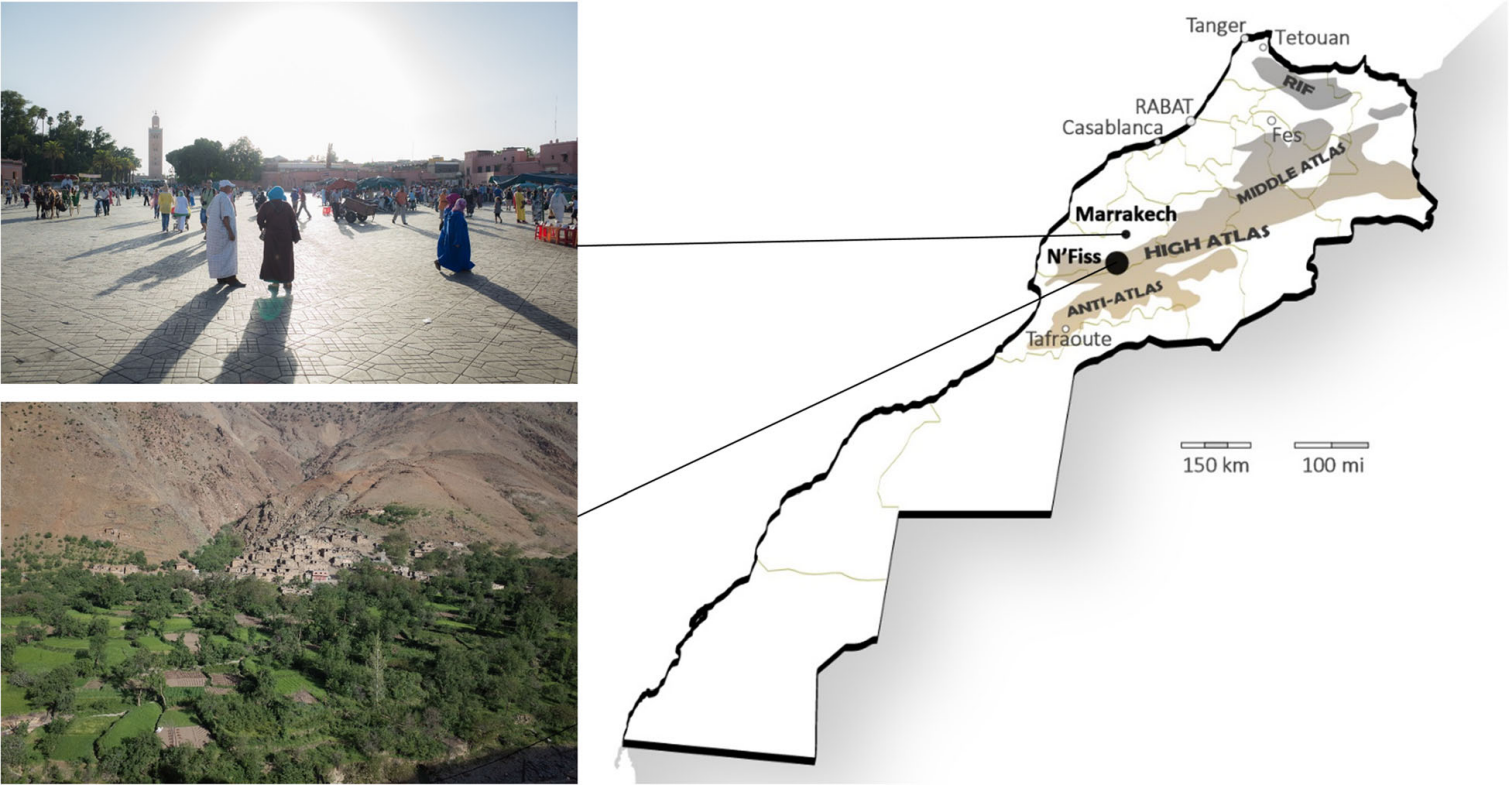
Map of Morocco and study sites: Marrakech and High Atlas in the N’Fiss valley

Morocco neighbours Algeria in the east and north-east and Mauritania in the south and south-east and has Atlantic and Mediterranean coastlines. Four mountain ranges, the Rif, the Middle Atlas, the High Atlas and the Anti-Atlas, form a semi-circle around the coastal and middle plains separating them from the Saharan desert (Fig. 1). This topography and the diversity of biomes found in the country favour botanical diversity, and the mountainous areas are considered biodiversity hot-spots within the Mediterranean [33]. Morocco has been at the cross-roads of ancient trade routes and its current population is the result of several waves of migration from Southern Europe, Arabia and Sub-Saharan Africa mixed with the indigenous North African populations [34].

French colonialism brought Western technology to Morocco at the beginning of the 20^th^ century to promote economic development, impacting agricultural and industrial production methods, and human and veterinary medical care. Economic development continued after independence, especially in urban areas since rural societies were viewed as highly conservative, hindering the strategic modernising goals of the new country [35]. Since the 1970s, the gross national income per person has quadrupled, the life expectancy increased by almost 20 years, the number of births per women decreased from six to two, approximately, and primary school net enrolment now reaches 95% [36]. The rise of a more orthodox form of Islam and compulsory state schooling are influencing a generational cultural change [37].

The present study was situated in the south of the Atlantic plains and neighbouring montane areas, both in the city of Marrakech and the mountainous areas of the El Haouz province, specifically in the N’Fiss valley in the High Atlas mountains. Marrakech and its metropolitan area now have more than a million inhabitants, many of them migrants from neighbouring rural areas, combining traditional and cosmopolitan lifestyles (Fig. 1). Different medical systems are available in Marrakech; renowned for its traditional healers, there are also modern private medical facilities, and everything in between. The rural communes where we conducted the study are directly south of Marrakech, in the High Atlas mountains (Fig. 1). Most of the population is Ishelhin, the indigenous ethnic group of Southern Morocco, who speak Tashelhit, an Amazigh (or Berber) language. According to a recent census approximately 10% of men also speak Moroccan Arabic [38] and younger generations are increasingly bilingual because they learn Moroccan and Classic Arabic at school and are more likely to participate in the market economy, where Moroccan Arabic is prevalent. Subsistence agriculture and pastoralism remain the main livelihoods in the High Atlas, contributing to three quarters of the local income [39]. Other income is generated by seasonal labour in urban areas, and specialized local occupations including shop keeping, mining and limited engagement in tourism. Biomedical facilities and resources are scarce: there is only one public health centre in each rural commune, for between 5000 and 8000 people [22]. Not all the public health centres have a doctor, and they are usually staffed by non-locals who do not speak Tashelhit, which can pose serious communication issues between staff and their patients.

## Methods

Research was performed following the guidelines of the American Anthropological Association [40] and the code of ethics of the International Society of Ethnobiology [41], and in accordance with the Declaration of Helsinki. Approval from the ethics committee of the School of Biological Sciences, University of Reading, was obtained (Research Ethics Project Submission SBS14-15 05). Before all interactions, prior informed consent was obtained. Unique codes were used to identify informants, whose names were not recorded in the survey to ensure anonymity. Oral permission for publication was obtained from informants. This study was carried out in Marrakech and rural areas of the High Atlas (N’Fiss valley) from February to May 2015. A mixed methods approach was used. All interviews were carried out by the first author.

The rural study focused on the commune of Imegdale, where we interviewed mothers that bring their infants to *ferraggat*, but it extended to five more rural communes, where some of the *ferraggat* lived and practised. Nine *ferraggat* from eight villages in five different rural communes were interviewed. Mothers were interviewed in 11 villages and selected randomly (*n* = 33). Healers (*n* = 13) were selected by asking mothers which *ferraggat* they normally visit or if they knew of any other women who practised *frigg*. In Marrakech, *ferraggat* were contacted through the extended social networks of research assistants. Seven *ferraggat* were contacted but three did not wish to participate in the study either because of unwillingness to talk while practising (they were the busiest *ferraggat* we encountered) or distrust. Four *ferraggat* were interviewed in Marrakech, including two who are originally from the High Atlas. In the N’fiss valley, all available public health professionals (*n* = 5) were interviewed in the health centres of Imegdale and Ouirgane. Since *frigg* is based on a recipe of medicinal plants and herbalists are the main herb suppliers when *ferraggat* do not harvest the plants themselves, we also interviewed herbalists (*n* = 10). Herbalists were selected randomly and interviewed in rural market places in the N’Fiss valley (Asni and Talat N’Yakoub) and Marrakech.

*Ferragat* (*n* = 13) and herbalists (*n* = 10) were asked to list the plants used in *frigg* (free-listing) [42]. Informants were also asked about where plants are acquired, whether the mixture always has the same ingredients and about criteria for plant selection. A quantitative approach was used to analyse free-lists by calculating use values (UV) for plants in *frigg* to quantify their cultural importance, following the equation UV = ∑Uis / N, where *∑Uis* is the sum of the total number of use reports concerning a given species and *N* is the total number of informants [43]. We used R v. 3.2.3 [44] to carry out a non-metric Multidimensional Scaling (MDS) analysis to visualise differences among the plant lists given by informants (*isoMDS* and *metaMDS* functions from the MASS and vegan libraries) [45, 46], and a t-test to evaluate differences in the number of plants listed by *ferraggat* and herbalists.

Since very little was known on the *frigg* subject, qualitative inductive methods where no hypothesis is pre-specified were used to elucidate the explanatory models [28], enquiring about the ailments treated, their symptoms, and disease aetiologies. Moreover, *ferraggat* were questioned about where and from who they had acquired this knowledge and if they had taught it to someone else. The same questions were asked of herbalists (*n* = 10). Moreover, we observed twelve healing sessions with five different *ferraggat*. We asked staff from public health centres if they knew about *frigg* and, in the case that they did, they were asked to describe what *ferraggat* do and to give their opinion on the practice. Mothers were asked if they brought their children to *ferraggat* when they were sick and why. Interviews were carried out with the assistance of a local Tashelhit-speaking translator in rural areas and a local Moroccan Arabic-speaking translator in urban areas. Qualitative data from in-depth interviews were analysed by cross-checking, summarising and synthesising data collected from interviews to construct a narrative account [47].

Plant specimens were collected in the field with the community’s permission, and preferably with the collaboration of an informant, or acquired in the nearest market place and vouchered. Authorisation for plant collection was granted from the University Cadi Ayyad, Marrakech, in accordance with national guidelines and legislation. A. Ouhammou and I. Teixidor-Toneu identified the specimens using the *Flore Pratique du Maroc* [48]; nomenclature and family assignments follow The Plant List [49]. Vouchers were deposited in the Marrakech Regional Herbarium (Morocco; MARK) and the University of Reading Herbarium (United Kingdom; RNG); permission to export voucher duplicates was obtained from the Faculty of Sciences Semlalia, University Cadi Ayyad, Marrakech.

## Results

### *Frigg* narratives

‘Doctors are good, but we need *frigg*’ (Mother in Imegdale)

*Frigg* is an important resource for infant healthcare in rural Morocco; 94% of the rural mothers interviewed seek help from *ferraggat* when their children are sick. According to the informants, *ferraggat* deal with ailments that biomedicine is not effective in treating because of their supernatural cause, often associated with sorcery. Mothers from isolated villages also mentioned accessibility and availability as an important reason to prefer traditional medicine. Public health centres are often far away, understaffed or under-resourced, or economically inaccessible, whereas *ferraggat* live nearby or travel to the patient when necessary. They work “*fi sabilillah*”, for the sake of Allah, without expecting compensation apart from donations. *Ferraggat* affirmed that they send infants to be treated in the hospital when the ailment is outside of their expertise. All workers of public health centres interviewed were opposed to *frigg* practise, characterising it as dangerous and backward.

We did not interview mothers in urban areas, but *ferraggat* do not lack patients in Marrakech. As they explained, mothers often bring their infants when biomedical treatments have proven ineffective. *Ferraggat* added that they also treat infants from “conservative” mothers who prefer traditional medicine because of their beliefs. *Ferraggat* in Marrakech are very much like those in the mountains in terms of the settings of the practice (generally their homes) and their availability (working “*fi sabilillah*”). However, it is worth mentioning the case of a very young *ferragga* who had an established practise in Marrakech with time schedules and set prices, showing a shift towards professionalization [50, 51].

### Ailments treated: reported symptoms and perceived causes

‘*Taumist* is a sickness for *ferraggat* to heal’ (*Ferragga* in Marrakech)

Three main paediatric ailments are treated by *ferraggat*: *taqait*, *taumist* (*sarra* in Moroccan Arabic) and *iqdi* (*shem* in Moroccan Arabic). They can also treat physical development and musculoskeletal problems and women’s ailments (not explored here). *Taqait* literally means “little globule” and the word is normally used to refer to unripe fruits. This word is used because diagnosis is normally made by checking the palate of the infant with the thumb; *ferraggat* consider the infant has *taqait* if she feels a little globule on the palate, “like a grain of corn”. Infants affected by *taqait* do not breastfeed, have difficulties in swallowing, can have ear pain (which *ferraggat* check by touching the area around the ears or softly pulling them) and may also be vomiting. The symptoms of *taqait* seem to correspond roughly to those of tonsillitis and ear infections. Workers from health centres showed no knowledge of this ailment and it was not mentioned by any *ferraggat* in Marrakech, so it could be a local folk illness from the High Atlas.

*Taumist* and *iqdi* are related, both are associated with a sorcery-related cause. *Taumist* means “bundle” and it refers to the talismans that people (normally women) carry. These talismans may heal or prevent disease, protect against the Evil Eye, or bring good luck. They are prepared by the *fqih*, the spiritual religious healer, part of the traditional therapeutic system [20]. They are often made from a paper wrap on which Quranic verses are written and contain salt and seeds of *harmel* (*Peganum harmala*). They may contain other plants such as *kzbor* (*Coriandrum sativum*), *sanouj* (*Nigella sativa*), *fijl* (*Ruta montana*) and *azuka* (*Tetraclinis articulata*). Babies can fall sick of *taumist* when around a person carrying a talisman. They are diagnosed with *taumist* when they have a sunken fontanel, extending to the forehead, the eyes may be rolling up, lethargy may be experienced and the skin has a greenish colour. They may also suffer from diarrhoea, abdominal pain, fever, or a combination of these symptoms. *Iqdi* literally means “to smell” and it refers to infants “smelling” sorcery or physical bad smells and consequently getting sick. The symptoms of *iqdi* are a bad skin smell, vomiting, diarrhoea and difficulty with breastfeeding. From a biomedical perspective, these two ailments seem to roughly refer to gastroenteritis, a very common condition in rural areas mostly due to poor diets and inadequate sanitation [52].

These traditional classifications of disease aetiology show both naturalistic and personalistic causes. According to Foster [15], personalistic aetiologies describe ailments caused by “the *active, purposeful intervention* of an *agent* who may be human (a witch or sorcerer), nonhuman (a ghost, an ancestor, an evil spirit), or supernatural (a deity or other very powerful being)” (p. 775, italics in the original), whereas naturalistic aetiologies “explain illness in impersonal, systematic terms”. *Taumist* and *iqdi* are not often the result of purposeful supernatural interventions, being caused by exposure to the harmful “properties” of magical items such as talismans, to which infants are vulnerable. Belief in sorcery as an active cause of adult health problems is widespread across Morocco [11], as well as the belief that infants are vulnerable and passive victims of its harmful effects until the age of two or when they have all their teeth.

### Treatment and prognosis

“Praise be to God, the child will heal” (*Ferragga* in Marrakech)

Healing sessions start with the creedal statement “*bismillah*”, “in the name of God”, and some *ferraggat* will sprinkle salt on the infant. The *Tibb-ul-Nabbi* (“Medicine of the Prophet”) of Mahmud bin Mohamed al-Chaghhayni states “Begin with salt, for verily it is remedy for seventy diseases” [53]. Fumigants can also be used at the beginning of the session, especially burning dry stems of *henna* (*Lawsonia inermis*) or *marrut* (*Marrubium vulgare*) close to the infant so they inhale the smoke. *Taumist, taqait* and *iqdi* are identified and diagnosed as different ailments but they are generally treated in the same way. *Frigg*, a blend of dried plants, ground and mixed with olive oil, is used to massage the baby’s body. It can also be administrated orally and sometimes as ear and nose drops. Mothers may bring the infant to the *ferraggat* just once or up to 3 days in a row, depending on the *ferraggat*’s recommendation or on the perceived effectiveness of the treatment. Finally, most *ferraggat* use *qtran rqeq* (lit. “thin cade oil”), oils extracted from the roots and branches of various *Juniperus* species, such as *J. oxycedrus*, and *Tetraclinis articulata*. A bit of the smoky, strong-smelling oil is put under the baby’s nose as well as on top of the head helping “clean out” the “bad smells” and protect the infant from further “smelling”. Although sessions usually proceed in this way (for a detailed description see Additional file 1), we interviewed one *ferraggat* who only used onion, salt and her inherited *baraka*. We were present in one of her healing sessions that started with the indispensable “*bismillah*” and sprinkling salt on the infant. Placing the infant on her lap, she took a piece of onion and rubbed it on the infant’s head, specifically the top and the sides of the forehead. She finally placed it on the top of the forehead and tied it with a piece of cloth. Ultimately, regardless of the treatment used, all *ferraggat* attribute healing to God’s will.

Perhaps surprisingly, herbalists reported an alternative mode of administration of plants by *ferraggat* and only two seemed familiar with *frigg* as reported by *ferraggat* themselves (Table 1). According to herbalists, plants used by *ferraggat* are put in a bundle of cotton cloth and infused in hot water. A bit of this water is given to the baby, usually by squeezing some drops from the bundle into the baby’s mouth. They call this mixture *taktira*, literally “drops” in Moroccan Arabic.

**Table 1:** *Frigg* practise and its transmission per informant. Informants marked with * were not included in the quantitative analysis (MDS)

Practitioner code, location (U = urban, R = rural)	Age	Source of knowledge	Knowledge transmitted to younger generations	Years of practise	Number of plants used	Source of plants used	Mode of preparation; mode of administration
Ferragga1, U	>70	Another *ferragga* in Marrakech	No	>40	12	Urban herbalists and harvested from the wild in the high Atlas	Dried ground plants, mixed with olive oil; oral ingestion and massage
Ferragga2, U	>80	Mother (family women’s lineage)	No	>40	6	Urban herbalists	Dried ground plants, mixed with olive oil; oral ingestion
Ferragga3, U	≈50	Mother	-	7	8	Urban herbalists	Dried ground plants, mixed in olive oil; oral ingestion and massage
Ferragga4, R	≈50	Mother	No	≈10	19	Harvested from the wild and cultivated in home garden	Dried ground plants, mixed with olive oil; oral ingestion
Ferragga5, R	>70	Another *ferragga* in Talat N’Yakoub	No	>40	7	Rural herbalists and harvested from the wild	Decoction in olive oil, filtered; nose drops and oral ingestion
Ferragga6, R	>70	Another *ferragga* in Tamslouht	-	>20	9	Rural herbalists and harvested from the wild	Dried ground plants, mixed with olive oil; oral ingestion
Ferragga7, R	≈50	Mother-in-law	No	≈30	10	Rural herbalists and harvested from the wild	Dried ground plants, mixed with olive oil; oral ingestion and massage
Ferragga8*, U	≈50	Father	No	>20	1	-	Topic
Ferragga9, R	>80	Mother	No	>60	9	Rural herbalists and harvested from the wild	Dried ground plants, mixed with olive oil; oral ingestion, ear and nose drops, massage
Ferragga10, R	≈50	Another *ferragga* in Maregha	No	3	16	Rural herbalists and harvested from the wild	Dried ground plants, mixed with olive oil; oral ingestion and massage
Ferragga11, R	>80	Mother-in-law	No	≈20	16	Rural herbalists and harvested from the wild	Dried ground plants, mixed with olive oil; massage
Ferragga12, R	>70	Sister-in-law	No	>20	12	Urban herbalists	Decoction of plants in olive oil, filtered; oral ingestion and massage
Ferragga13*, R	≈60	Mother-in-law	Yes	10	-	Urban herbalists	Dried ground plants, mixed with olive oil; massage
Herbalist1, U	≈40	Father	-	18	15	Wholesalers	Dried plants infusion; oral ingestion
Herbalist2, U	28	Father	-	5	7	Wholesalers	-
Herbalist3, U	85	Another herbalist in Marrakech	-	48	13	Wholesalers	Dried plants infusion; oral ingestion
Herbalist4, U	≈40	Father and other herbalists in Marrakech	-	>20	19	Wholesalers	Dried plants infusion; oral ingestion
Herbalist5, U	42	Father	-	32	22	Wholesalers	Dried plants infusion; oral ingestion
Herbalist6, U	≈50	Father	-	>30	11	Wholesalers	Dried plants infusion; oral ingestion
Herbalist7, U	≈50	Father and elder brother	-	>30	16	Wholesalers	Dried plants infusion or mixed with olive oil; oral ingestion, ointment for massage
Herbalist8, R	≈60	Other herbalist in Marrakech	-	≈40	9	Wholesalers and collectors (harvested from the wild)	Dried plants infusion; oral ingestion
Herbalist9, R	≈30	Grandfather	-	11	9	Wholesalers	Dried plants infusion; oral ingestion
Herbalist10, U	≈50	Father	-	>20	12	Wholesalers	Dried plants infusion; oral ingestion

Musculoskeletal and development problems are treated by various stretching exercises and thorough massage with either *frigg* or a cream acquired in the pharmacy (in urban areas), followed by specific bundling sometimes with the use of wooden boards as supports. *Qwi*, a traditional cauterisation method, is sometimes also used to treat musculoskeletal problems. A burning, dried stem of *marrut* (*Marrubium vulgare*) will be used to lightly touch specific points of the baby’s body, especially around joints, the abdominal area and the back. Unlike this practise in other countries [54], the stick touches the infant’s skin very lightly and it was reported that the treatment never leaves a mark.

### Ingredients of *frigg*

“If a baby gets sick, I give him herbs” (*Ferragga* in Talat N’Yakoub)

A high diversity of plants is used in *frigg*. In total, we inventoried 67 vernacular plant names corresponding to 70 botanical species. Five vernacular names were generic complexes (referring to more than one botanical species) and two others could not be identified. Informants listed 12 (±4) plants on average, and the number of plants listed by *ferraggat* or herbalists was not significantly different (t-test, *P*-value > 0.05). Fifteen vernacular names were only mentioned by one informant; plants mentioned by two or more informants are listed in Table 2. Almost half of the plants belong to three families: Lamiaceae (26%, 17 species), Apiaceae (9%, six species) and Asteraceae (8%, five species). Plants with the higher use values are *karwiya* (*Carum carvi*; 0.64), *timija* (*Mentha suaveolens*; 0.59), *azukni* (*Thymus saturejoides*; 0.59), *kamun sofi* (*Ammodaucus leucotrichus*; 0.55)*, rman amrouj* (*Punica granatum*; 0.55), *khzema* (*Lavandula angustifolia*; 0.50), *zaʕter* (*Origanum compactum*; 0.45), *fliyou* (*Mentha pulegium*; 0.45), *uamsa* (*Foeniculum vulgare*; 0.45), *lwerd* (*Rosa sp.*; 0.45) and *sanouj* (*Nigella sativa*; 0.41). Except for *kamun sofi*, these are all commonly used plants in the High Atlas [18]. Informants describe medicinal plants used in *frigg* as “mild”, “not bitter” or “not spicy” (*maharrsh* in Moroccan Arabic); “bitter” plants (*harr* in Moroccan Arabic) such as *shndgora* (*Ajuga iva*) and *shih* (*Artemisia herba-alba*) are always used in small doses*.*

**Table 2:** Botanical identification of the plants used in *frigg*, voucher specimen, number of use reports mentioned by *ferraggat* (URf) and herbalists (URh), and use value. The phonetic symbol ʕ has been used to designate the Arabic letter ‘ayn (ع)

Family	Vernaculars	Species (Voucher number)	Plant part used	URf	URh	UV
Amaranthaceae	mkhinza	*Dysphania ambrosoides* (L.) Mosyakin & Clemants (IME02)	Leaves	2	0	0.09
Apiaceae	habt halawa	*Pimpinella anisum* L. (MAR40)	Fruits	1	6	0.32
	kamun beldi	*Cuminum cyminum* L. (MAR66)	Fruits	3	0	0.14
	kamun sofi	*Ammodaucus leuchotricus* Coss. (MAR41)	Fruits	4	8	0.55
	karwiya	*Carum carvi* L. (MAR38)	Fruits	5	9	0.64
	kzbor	*Coriandrum sativum* L. (IME42)	Fruits	4	0	0.18
	n*ʕ*fa, uamsa	*Foeniculum vulgare* Mill. (IME27)	Fruits	2	8	0.45
Asteraceae	babunj	NA (NA)	Flowers	2	3	0.23
	itzghi, ifski n’uarras	*Cladanthus scariosus* (Ball) Oberpr. & Vogt (IME34)	Aerial parts	3	0	0.14
	jaidia, l*ʕ*ggaye	NA (MAR36)	Flowers	0	2	0.09
	shih	*Artemisia herba-alba* Asso (IME17)	Aerial parts	4	3	0.32
Brassicaceae	habrrchad	*Lepidium sativum* L. (MAR69_14)	Seeds	1	3	0.18
Cactaceae	ajdig n’ouknari, nuwrat lkarmus	*Opuntia ficus-indica* (L.) Mill. (IME100)	Flowers	2	4	0.27
Cistaceae	irgl	*Cistus salviifolius* L. / *Cistus creticus* L. (IME56 / IME86)	Leaves	1	1	0.09
Cupressaceae	azuka, l*ʕ*r*ʕ*r	*Tetraclinis articulata* (Vahl) Mast. (IME07)	Leaves	3	0	0.14
Fabaceae	helba, tifidas	*Trigonella foenum-graecum* L. (IME60)	Seeds	1	1	0.09
Iridaceae	za*ʕ*fran	*Crocus sativus* L. (NA)	Stigmas	4	0	0.18
Lamiaceae	azir, liazir	*Rosmarinus officinalis* L. (MAR48)	Leaves	2	5	0.32
	fliyou	*Mentha pulegium* L. (IME39)	Leaves	5	5	0.45
	grzguiel	*Lavandula maroccana* Murb. (IME06)	Leaves	2	0	0.09
	ifzi, lmrrut	*Marrubium vulgare* L. (IME24)	Leaves	4	0	0.18
	khzema	*Lavandula angustifolia* Mill. (MAR5)	Leaves, inflores.	6	5	0.50
	menta	*Clinopodium nepeta* (L.) Kuntze (MAR1)	Leaves	1	5	0.27
	shndgora	*Ajuga iva* (L.) Schreb. (IME68)	Leaves, flowers	1	2	0.14
	timija	*Mentha suaveolens* Ehrh. (IME05)	Leaves	7	6	0.59
	timzurria, imzurri	*Lavandula dentata* L. (IME03)	Leaves, inflroes.	4	0	0.18
	z*ʕ*tra, azukni	*Thymus saturejoides* Coss. (IME37)	Leaves, inflores.	5	8	0.59
	za*ʕ*ter	*Origanum compactum* Benth. (MAR14)	Leaves	2	8	0.45
Linaceae	zare*ʕ*t lktan	*Linum* sp*.* (NA)	Seeds	1	2	0.14
Lythraceae	rman amrouj	*Punica granatum* L. (IME61)	Flowers	4	8	0.55
Myrtaceae	knorfel	*Syzygium aromaticum* (L.) Merr. & L.M.Perry (MAR49_06)	Fruits	2	1	0.14
	riham	*Myrtus communis* L. (MAR22)	Leaves	1	2	0.14
Nitrariaceae	harmel	*Peganum harmala* L. (IME101)	Seeds	3	1	0.18
Pedaliaceae	jnjlan	*Sesamum indicum* L. (NA)	Seeds	2	1	0.14
Piperaceae	l*ʕ*sfor, bzar	*Piper nigrum* L. (MAR49_05)	Fruits	0	2	0.09
Ranunculaceae	sanouj	*Nigella sativa* L. (MAR8)	Seeds	3	6	0.41
Rosaceae	werd	*Rosa* sp*.* (IME105)	Flowers	4	6	0.45
Rutaceae	fijil, aurmi	*Ruta chalepensis* L. / *Ruta montana* (L.) L. (IME73 / MAR6)	Leaves	3	0	0.14
Schisandraceae	badiana	*Illicium verum* Hook.f. (MAR49_17)	Fruits	1	1	0.09
Verbenaceae	louisa	*Aloysia citridora* Palau (NA)	Leaves	0	3	0.14

Various opinions about the importance of using plants were found among *ferraggat*. Some informants thought that correct dosages and the good quality of ingredients were the sole key to a treatment’s success. Other *ferraggat* believed plants are good but the *ferragga*’s touch is more important for the treatment to work. This explanation is in line with what mothers believed about the treatment. Interestingly, eight informants (including three *ferraggat*) recalled that as recently as one generation ago, fewer plants were used or plants were not used at all. Seven colours of wool yarn and blood from a sheep slaughtered during *Eid Al-Adha*, one of the two main Islamic festivities, had been used to treat these same infant ailments. This practice is not carried out in the present.

**Fig. 2:**
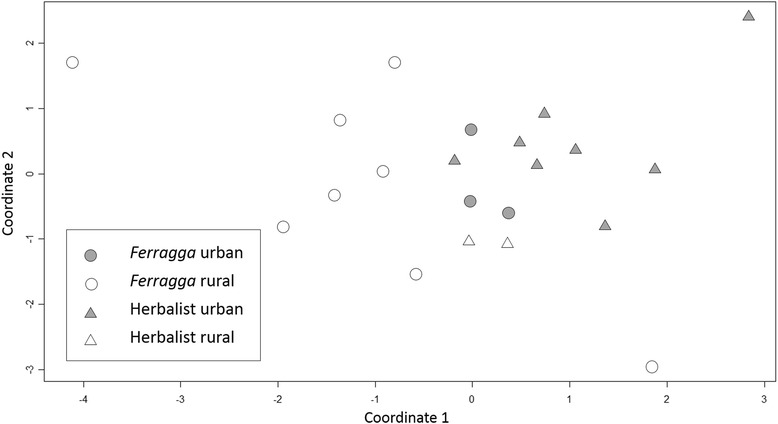
Multidimensional Scaling analysis of plant lists given by *ferraggat* and herbalists (stress factor 0.19)

As mentioned above, *ferraggat* and herbalists often give the medicinal plant mixture different names (*frigg* versus *taktira*) and the composition of the mixtures used or sold is also different (Fig. 2; MDS stress factor = 0.19). Mixtures used by urban *ferraggat* are more similar to those known and sold by herbalists because these two groups have the same medicinal plants available, i.e. those that are traded. Lists from rural *ferraggat* include many plants that grow in the High Atlas and are not usually traded, such as *mkhinza* (*Dysphania ambrosoides*), *ifski n’uarras* (*Cladanthus scariosus*) and *grzguiel* (*Lavandula maroccana*). However, *ferraggat*’s lists include *kzbor* (*Coriandrum sativum*), *azuka* (*Tetraclinis articulata*) and *zfran* (*Crocus sativus*), all traded species not present in herbalists’ lists. Although herbalists do not act as healers in this case, their knowledge is not negligible, since all *ferraggat* rely on at least some traded plants and some acquire ready-made mixtures (Table 1). Differences in herbalists’ mixtures compared to those used by *ferraggat* could also be biased by the herbalists’ wish to sell specific plants and lack of knowledge about untraded plants that are common in the High Atlas.

### Transmission of knowledge about *frigg*

“I learnt from my mother who learnt from her mother” (*Ferragga* in Imegdale)

Knowledge entails being aware of facts as well as knowing how to put them into use [55]. In the case of *frigg*, facts include which plants to use, where to acquire them, which ailments to treat, their symptoms and how to diagnose them, and skills encompass knowing how to prepare the herbal remedies, handle the infant and deliver the treatment. Knowledge about the medicinal plants used in *frigg* is transmitted orally and most *ferraggat* had learnt from their mothers, grandmothers or mothers-in-law (vertical and oblique transmission, sensu Cavalli-Sforza and Feldman [56]; Table 1). Others had learnt from another *ferragga* (horizontal transmission). Herbalists learnt mainly from their fathers (80%, vertical transmission) and occasionally also from elder herbalists to whom they were apprenticed (oblique transmission; Table 1).

Unlike other folk knowledge about medicinal plants that people learn continuously from childhood to adulthood or need to come in direct contact with natural environments [57], learning about *frigg* occurs when women are middle aged and already have children. *Ferraggat* had learnt either from a relative or from a peer. When learning occurs from a relative, women observe and help elder *ferraggat* practise at home for long periods of time. On the other hand, *ferraggat* that had learnt from a peer explained that there is actually no teaching on how to become a *ferraggat* besides a few indications, such as the list of medicinal plants used and how to diagnose. Other *ferraggat*, herbalists, knowledgeable older women and even pharmacists were mentioned as sources of knowledge about medicinal plants. Only one of them reported she had gone through a period of apprenticeship. Importantly though, in all cases permission and the gift of *baraka* has to be given from another *ferragga* for a woman to become a *ferragga* herself. This gift and transfer of authority is sometimes materialized by a pinch of salt that is physically given, and will be kept and passed on. Only two *ferraggat* considered this unimportant and placed much more value on the plants themselves. Four *ferraggat* explained that they became healers unintentionally: when learning how to treat their own infants, they were passed the gift of *baraka* from another *ferragga* and thus they accepted the moral obligation to treat other infants if mothers in need approached them.

At the time of this study, only one *ferragga* had already taught someone else, but this apparent lack of transmission to younger generations is partially misleading. Most *ferraggat* only started learning and practising when there was the perceived need for it, for example when the village *ferragga* was dying or moving out, so the *ferraggat* we interviewed may still pass on their knowledge. Also, mothers that learn how to treat their children without the intention of becoming a *ferragga* may become healers in the future. However, many of *ferraggat* explained that young people are not interested in learning about traditional medicine, a view that was shared by mothers. When discussing the possible lack of *frigg* specialists in the future, several informants were of the opinion that ailment-related beliefs will also change so people may just go to the hospital.

## Discussion

Cultural and socioeconomic background are well known factors driving health care seeking behaviour [31, 58–60]. In Morocco, biomedical treatment of illness predominates: a high proportion of patients use only biomedical resources or combine traditional and biomedicine [11, 32]. According to Obermeyer [32], Moroccan women’s beliefs and healthcare practices during pregnancy and birth allow a coherent integration of traditional and biomedical practice. As for other folk ailments with personalistic aetiologies [61], including illnesses resulting from the Evil Eye [62], popular explanatory models for *taumist* and *iqdi* demand the use of traditional specialists. *Ferraggat* address illnesses in a context of shared beliefs about health and ailment causality, i.e. the contact of the child with sorcery. They are associated with Baraka, or divine blessing as the gift for healing, so perceived efficacy of *frigg* may be influenced by cultural constructs of efficacy [20, 24] and responses to meaning [25]. Equally important, *ferraggat* in rural areas have personal relationships with their patients and few time constraints, so psychological aspects of healing can be enhanced, contributing to the perceived efficacy of their therapy [27, 63].

*Frigg* includes a mixture of ritualistic and ethnopharmacological treatments, as is common for other folk ailments with personalistic aetiologies [29, 61]. In the case of *susto*, phytochemical activity of oral plant remedies has been demonstrated [64] and yet these are not the most common form of treatment and are often used externally [29]. Species-rich mixtures such as *frigg* may be effective means to treat ailments with multifactorial causes, including gastroenteritis [65], and there is evidence for pharmacological activity for over half of the high use value plants used in *frigg* [18]. However, narratives about the *frigg* practice suggest that specific plant species are not as important to achieve healing as the healing hand itself. We evidence recent incorporation of plants into this healing practise, replacing more ritualistic treatments. The shift to plant based remedies used in *frigg* is probably due to currently popular narratives on plant efficacy and the influence of Islamic reforms. Symbolic remedies such as coloured wools and blood are linked to pre-Islamic beliefs, nowadays considered *haram* practises forbidden by Islam. Herbal remedies are a currently accepted means of treatment, representing the fusion of nature with science [66], and are acknowledged by religion [53]. The rise of a more orthodox Islam, enhanced religious education in rural areas and globalisation, are also altering other local beliefs and practises in the High Atlas such as saint worship [67] or women’s tattooing (Teixidor-Toneu et al., *in prep.*). Islam has also been observed to affect the symbolic framework of healing practices in other Muslim countries [30]. Traditional therapeutic systems where traditional healers practice are dynamic, historically contingent, and embedded within social institutions and socioecological processes. Thus, practices and treatments can be influenced by contact with modernity, science and technology, as well as by religious reforms. Although, medicinal plant use as a means of healing is often viewed as the result of indigenous experimentation with the environment over centuries (see for example [57]), there is evidence for rapid change of herbal practises when isolated indigenous groups come in contact with new cultures and plants. For example, the Ese Eja elders testify that in recent memory healers cured without using or ingesting any plants; in this context the adoption of plants represents the acquisition of knowledge, power and agency from “outsiders” [68]. Similarly, Polynesians are thought to have had a limited herbal medicine tradition prior to European contact [69]. The use of mixtures is ubiquitous in the High Atlas and Morocco in general [13, 18], and our analysis suggests that mixtures of widely-available plants are easy to adopt and function to legitimise the *ferraggat*’s practice in the context of modernisation and biomedical treatment, by a transference of cultural meaningfulness from currently illicit symbols. Indeed, ritualistic elements from the *frigg* practice that are accepted by religion, namely the use of salt, are maintained.

Sociocultural changes can result in changing experiences of illness and their explanatory models, and acculturation can lead to their dismissal altogether [70]. Although we did not observe changes in the conceptualisation of the studied culture-specific ailments because of strong beliefs in supernatural ailment aetiologies, schooling and exposure to biomedicine could have an effect of this kind in the near future. School attendance reduces time at home learning about health, or outdoors acquiring empirical ecological knowledge [71, 72] and presents a discourse where village-based knowledge is perceived as backward [73]. This goes hand in hand with the narratives on the supremacy of biomedicine from healthcare professionals, effectively shifting local medical knowledge paradigms. Leonti and Casu [65] predict that changes in explanatory models could reduce the effectiveness of traditional therapies, because their meaning is weakened. *Frigg* practice altogether could also experience a loss of meaning in future generations, as observed for *eghindi*, a culture-specific ailment among the Sahrawi [70]. Whether we are documenting a differential change in different aspects of medicinal practice, with disease conceptualisation persisting whilst the object of meaning for treatment adapts, should be the focus of future research. This could provide insights into the processes by which traditional medical systems adapt to sociocultural, economic and environmental changes.

## Conclusion

We have presented a current overview of a traditional medical treatment, *frigg*, a practice not previously reported in the ethnomedical literature, in the context of modernisation and contact with biomedicine. *Frigg* is used to treat mostly ailments with personalistic causes, and this drives mothers’ to seek treatment from traditional healers who practise in a context of shared beliefs on health and illness. The treatment has changed, and biomedicine is currently available, but preference of mothers to visit *ferraggat* for certain infant ailments persists, as observed for other illnesses in various cultures. Contrary to the widespread view among ethnobotanists that medicinal plant knowledge is archaic and largely based on prolonged experimentation in one’s environment, we report that widely available medicinal plants as the core for treatment might be recently adopted. Southern Moroccan women and healers maintain folk conceptualisations of illness, but adapt treatments to match modern ideas, especially those stemming from new religious viewpoints. Use of medicinal plants is historically contingent and may serve to legitimise a treatment which a generation ago relied on apparently more symbolic remedies. This case study provides new insights on the dynamic aspects of traditional medicine and how sociocultural changes impact medicinal plant use, challenging widespread views about medicinal plant use being archaic.

## Additional file

Additional file 1: Detailed description of a *frigg* session in Marrakech from I.T.’s field journal. (DOCX 15 kb)
