# Supplementary material for: Treating infants with frigg: linking disease aetiologies, medicinal plant use and care-seeking behaviour in southern Morocco
Source: J Ethnobiol Ethnomed. 2017 Jan 13;13:4. doi: 10.1186/s13002-016-0129-4 (PMC5237284; doi:10.1186/s13002-016-0129-4)
Supplement: Additional file 1: — Detailed description of a frigg session in Marrakech from I.T.’s field journal. (DOCX 15 kb) [file 13002_2016_129_MOESM1_ESM.docx]

**Additional file 1**

Detailed description of a *frigg* session in Marrakech from I.T.’s field journal.

In the sitting room of the *ferragga*’s house, in Marrakech, we are discussing the medicinal plants she uses when someone knocks the door. Two women, one of them carrying a baby, come in and we all move to a small room where the *ferragga* receives her patients. Some blankets are laid on the floor and several pots with ground mixtures and plastic bags are placed on a little table in one corner, next to a gas burner. After the usual greetings and some small talk, the mother explains that her baby has fever and his chest is congested. She had been in the hospital, where his child was treated to get phlegm out from his chest, but the treatment (not specified) is not working. It is the first time that she visits this *ferragga*, but she had visited another *ferragga* fifteen years ago, when another of her children was sick. The second woman, the mother’s friend, had recommended this *ferragga* because she had heard she is good. The baby is crying and the mother mentions that he cries a lot, sometimes until he is breathless.

The *ferragga* takes the baby on her lap, says “*bismillah*” (“in the name of God”) and tries to make him smell the smoke from a dried burned *marrut* (*Marrubium vulgare*) stem. Since he is moving a lot, he goes back to his mother’s arms so she can hold him. The mother says “don’t bother fumigating him, just do the other…”, but her acquaintance interrupts “let her fumigate him, it is just *baraka*” [in Moroccan Arabic, *baraka* also means “enough”, and in this context the two meanings are expressed at once]. The baby is facing the *ferragga* and she fumigates him, while he tries to get away from the smoke. When she is done, she says “*ʕallah slamtek*” (the phonetic symbol ʕ has been used to designate the Arabic letter ayn, ع), which roughly translates into “Allah give you health”, but also denotes the end an event that took a long time or was arduous. Then, she asks if the mother brought clothes to change the boy into, since he may throw up. No fresh clothes had been brought, so the *ferragga* uses some towels and clean pieces of cloth she keeps in a bag to cover the baby’s chest.

The *ferragga* turns on the gas burner and places a little pot on top where she pours some olive oil and a spoonful of the *frigg* herbal mix. She turns the gas off when the mixture is just slightly warm. She says “*bismillah*” again and asks the mother to hand her the child so she can put him on her lap. The baby is crying loudly. She holds the baby on her lap and feeds him some of the mixture with a small spoon. The baby coughs up some of it. The *ferrgga* says “*beshefa inshallah*”, which is a wish for recovery from illness and then “*belʕfou el waldi*” which means “be spared [from illness] my child”. The *ferragga* keeps feeding him *frigg* and the child continues to cough it up. The *ferragga* repeats these expressions once again and explains that she is hoping the boy will throw up, which would help him. She mentions that the baby is feverish and the mother explains he has a cold, but the *ferragga* says it has to do with his teething. Then she checks the baby’s abdomen and comments it is inflated because he has been exposed to the cold too much. She partly undresses the baby and massages some of the remaining *frigg* on his abdomen, chest and back. Once he is dressed up again, she uses a bit of *qtran rqeq* (lit. “thin cade oil”) and rubs it with her fingers on top of the boy’s head and under his nose.

Finally, the *ferragga* wraps the baby with a thin blanket, making sure to cover his head, in a Moroccan traditional fashion called *gmat*. The mother comments the fact that he didn’t throw up during the session and the *ferragga* tells her not to worry, “it will leave his body when he defecates”. The *ferragga* advises to use orange blossom water to help reduce the fever by applying it to the baby’s head as it has cooling properties. She explains that the baby has cold and *shem* (*iqdi* in Tashelhit). The mother asks if she can feed her child and after the *ferragga*’s approval she does so. Some money is given to the *ferragga*, who doesn’t look at it and puts it in her apron’s pocket without looking at it. The mother and her acquaintance talk about getting a taxi so the child needs not to be exposed to the cold when they go back home and the *ferragga* gives the mother instructions on how to cover the child so they don’t need to worry when they wait for the taxi. The *ferragga* tells the mother not to be afraid for her child and that he will get better. She explains that infants come to her, drink the medicine, get massaged and go to sleep. And next day their mothers say they are fine, but come back to repeat the treatment. The mother asks if she has to bring her baby back since she lives so far away. The *ferragga* asks her if she can give the child some *frigg* just as she did and gives her some ground mixture and the instructions to give the baby a bit next morning and the afternoon, “that will be enough”.
